# Identification and Expression Analysis of BURP Domain-Containing Genes in *Medicago truncatula*

**DOI:** 10.3389/fpls.2016.00485

**Published:** 2016-04-13

**Authors:** Yuan Li, Xue Chen, Zhu Chen, Ronghao Cai, Hongmei Zhang, Yan Xiang

**Affiliations:** ^1^Laboratory of Modern Biotechnology, School of Forestry and Landscape Architecture, Anhui Agricultural UniversityHefei, China; ^2^Key Laboratory of Crop Biology of Anhui Province, School of Forestry and Landscape Architecture, Anhui Agricultural UniversityHefei, China

**Keywords:** abiotic stress, bioinformatics, BURP genes, *Medicago truncatula*

## Abstract

BURP domain-containing proteins belong to a newly identified protein class that is unique to plants and plays an important role in plant development and metabolism. Although systematic characterization of BURP domain-containing proteins have been carried out in many species, such as rice, poplar and maize, little is known about BURP domain-containing proteins in *Medicago*. In this study, multiple bioinformatics approaches were employed to identify all the members of BURP family genes in *Medicago*. A complete set of 39 BURP family genes were identified. These genes have diverse structures and were distributed on chromosome 1–8 except 7. According to phylogenetic analysis, these BURP family genes could be classified into eight classes. Motif and exon-intron organization, stress-related *cis*-elements in promoter regions and microarray analysis of *MtBURPs* were also performed. Furthermore, transcript level analysis of *MtBURP* genes in response to drought stress revealed that all of the 39 BURP genes were regulated by drought stress. The results of this study reveal a comprehensive overview of the *Medicago* BURP gene family and provide the first step toward the selection of *MtBURP* genes for cloning and functional analysis of the BURP gene family in *Medicago truncatula*.

## Introduction

The BURP domain is a conserved C-terminal protein domain whose name is based on four typical members, BNM2, USP, RD22, and PG1β. BURP domain-containing proteins have so far only been found in plants, suggesting that their functions may be plant specific. BNM2 is a protein specifically expressed during microspore embryogenesis in oilseed rape (Boutilier et al., 1994); USPs, which are expressed during the early stages of zygotic (Bassüner et al., 1988) and *in vitro* embryogenesis (Chesnokov et al., 2002) in field bean; RD22 is a dehydration-responsive protein in *Arabidopsis thaliana* (Yamaguchi-Shinozaki and Shinozaki, 1993), and PG1β, a non-catalytic β-subunit of the polygalacturonase isozyme 1, which is expressed during the ripening of tomato (*Solanum lycopersicum*) (Zheng et al., 1992).

BURP domain-containing proteins have several conserved modules: an N-terminal hydrophobic domain with a presumptive transit peptide; a short conserved segment or other short segment; an optional segment consisting of repeated units, which is unique to each member of the BURP-domain-containing family, and the C-terminal BURP domain (Hattori et al., 1998). BURP is a motif of approximately 230 amino acids and usually contains several highly conserved sequences, including two phenylalanine (F) residues, two cysteine (C) residues and four repeated cysteine-histidine (CH) motifs, and generally conforms to the sequence as CHXCHX_23-27_CHX_23-26_CHX_8_W (where X is any amino acid residue) (Ding et al., 2009).

BURP domain-containing proteins have been found in many species, such as rice, soybean, maize, and sorghum (Ding et al., 2009; Xu et al., 2010; Gan et al., 2011). A systematic analysis of BURP genes has been carried out in each of these species. Based on phylogenetic analysis of the putative BURP domain-containing proteins in soybean and other plant species, the members of the BURP family are classified into the BNM2-like, USP-like, RD22-like and PG1β-like subfamilies (Granger et al., 2002). Differences among the BURP domain-containing proteins mainly occur in a variable region between the hydrophobic signal sequence and the BURP domain (Ding et al., 2009). This region includes a short conserved segment and an optional segment consisting of repeated units which are absent in BNM2-like proteins. PG1β-like proteins are distinguished from the members of other subfamilies by the presence of a sequence segment containing a 14-amino-acid sequence (Zheng et al., 1992). Both the USP-like and RD22-like proteins are distinguished from other subfamilies by the presence of a sequence segment containing approximately 30 amino acids, but the RD22-like proteins have a segment consisting of repeated units of approximately 20 amino acids, while the USP-like proteins do not contain such a segment (Granger et al., 2002).

Many BURP proteins have been isolated from various plants species, but their expression patterns are diverse with some functions still unknown. BURP domain-containing proteins play an important role in maintaining normal plant metabolism or development (Shao et al., 2011; Tang et al., 2014). VfUSP, an abundant non-storage seed protein from field bean (*Vicia faba L.*) with unknown functions, is expressed during the early stages of zygotic embryogenesis (Bassüner et al., 1988) and *in vitro* embryogenesis (Chesnokov et al., 2002), respectively. PG1β, the non-catalytic β-subunit of the polygalacturonase isozyme (PG) from ripening tomato (*Solanum lycopersicum L.*), plays a significant role in regulating pectin metabolism by limiting the extent of pectin solubilization and depolymerization (Zheng et al., 1992; Liu et al., 2014). *ASG1* is a gene that specifically expressed during early embryo sac development in apomictic gynoecia of *Panicum* (Chen et al., 1999), but it is not expressed in sexual gynoecia of *Panicum* (Chen et al., 1999). *AtUSPL1* was found in cellular compartments such as Golgi cisternae, dense vesicles, prevaculoar vesicles and protein storage vacuoles in the parenchyma cell of cotyledons and thus may play a role in seed development (Tiedemann et al., 2009). BNM2-like was originally found to be expressed in microspore-derived embryos of *Brassica napus*, but recent studies have shown that BNM2-like is confined to seeds and is localized to the protein storage vacuoles (Treacy et al., 1997). The mRNA encoding *SCB1* (seed coat BURP-domain protein 1), is specifically found within the seed coat during the early stages of soybean seed development. *SCB1* helps regulate the formation of the soybean seed coat by governing the differentiation of seed coat parenchyma cells (Batchelor et al., 2002). *VvBURP1* belongs to the BURP domain-containing protein family. This protein is similar to the BNM2-like proteins, and the expression profiles of *VvBURP1* in different grapevine organs are close to those in soybean (Fernandez et al., 2007). *OsRAFTIN1*, an anther-specific protein in rice (*Oryza sativa L.*), transports sporopollenin from tapetum to developing microspores via Ubisch bodies (Wang et al., 2003).

Many BURP domain-containing proteins are also stress induced. The expression of RD22 is induced by drought, abscisic acid (ABA) and salt stress, and mechanisms underlying this regulation have been well-studied (Urao et al., 1993). Four genes from *Bruguiera gymnorrhiza* (*BgBDC1, 2, 3*, and *4*) encode BURP proteins are similar to RD22 and may function in responses to abiotic stresses such as drought stress and ABA induction, as well as biotic stresses such as plant vegetative and reproductive development, plant diseases and insect damage (Harshavardhan et al., 2014). The expression of *SALI3*-*2* and *SALI5*-*4a*, putative BURP protein genes, is induced by aluminum stress in soybean (Ragland and Soliman, 1997). The transcript level of *BnBDC1*, a shoot-specific gene encoding a BURP protein in oilseed rape, is up regulated by salt, ABA and osmotic stresses and down regulated by salicylic acid. Thus this gene may be involved in both abiotic and biotic stresses (Shunwu et al., 2004). The over expression of *SALI3*-*2* increases salt tolerance in yeast, but signal peptide deletion weakens salt tolerance (Ragland and Soliman, 1997). These reports suggest that BURP family genes might be crucial not only for plant development but also for response and adaptation to stresses.

*Medicago* is an annual flowering plant known as “the king of the forage grass.” Compared with other leguminous plants, the diploid *Medicago truncatula* is a fast-growing plant with a small genome, and it has therefore become a model plant for DNA sequence analysis (Endre et al., 2002; Stacey et al., 2006). However, *Medicago* production is threatened by drought and other environmental stresses. Although BURP genes have been systematically characterized in rice, maize and poplar, an extensive analysis of BURP genes in *Medicago* has not been reported. As the genome sequence of the *Medicago* is complete, it is possible to analyze the entire family of *Medicago* BURP proteins. In the current study, 39 putative genes of the BURP family were identified. To discover the functions of all the members, these genes were subjected to phylogenetic analysis, structural analysis, microarray analysis and expression profile analysis. In addition, the transcript levels of all 39 genes under drought stress were investigated. The results presented in this study showed that the expression of most of the *Medicago* BURP genes is non-tissue-specific but stress-responsive. This research will enable systematic analysis of the functions of the *MtBURP* gene family in the future.

## Materials and Methods

### *In Silico* Identification of Putative BURP Proteins in *Medicago truncatula*

First, the *M. truncatula* protein database was downloaded from http://www.medicagohapmap.org/home/view. The Hidden Markov Model (HMM) profile of the BURP domain (PF03181) was then obtained from the Pfam database^1^ (Finn et al., 2006; Ding et al., 2009). This HMM profile was then employed as a query to identify all BURP-containing sequences by searching BURP domain sequence against the *M. sativa* protein database using BlastP (*P*-value = 0.001). Finally, all of the primary candidates were identified by performing complete sequence alignment with MEGA v4.0 (Tamura et al., 2007) and checked manually to remove any overlapping sequences. And all of the non-redundant BURP genes were subjected to further analysis.

### Chromosomal Localization of *Medicago truncatula* BURP Genes

A chromosome location image of *M. truncatula* BURP genes was generated with MapInspect software ^2^ according to their starting positions on the *M. truncatula* chromosomes. The starting positions of *M. truncatula* BURP genes were obtained from http://www.medicagohapmap.org/genome. The genes were designated *MtBURP1* to *MtBURP39* according to the gene’s position, from the top to the bottom, on *M. truncatula* chromosomes (1–8).

### Phylogenetic Analysis

To study the genetic relationships among the BURP domain-containing proteins and infer the evolutionary history of this gene family, the multiple sequence alignment of all predicted *Medicago* BURP protein sequences was performed with Clustal X2.0 software using default parameters. Then, based on this alignment, phylogenetic trees were constructed using Clustal X2.0 with the Neighbor-Joining (NJ) method, and bootstrap analysis was conducted using 1,000 replicates (Hu et al., 2010). A phylogenetic tree was initially constructed using the complete *MtBURP* protein sequences and the full-length protein sequences from rice, sorghum, maize, soybean, 4 BURP proteins in *Arabidopsis* (*AT1G49320.1, AT1G70370.1, AT1G23760*, and *AT1G60390.1*) and 4 host BURP members (*AtRD22, Vf-USPs, BNM2* and *Le-PG1*β). Classification of the *MtBURP* genes was then performed according to their phylogenetic relationships with corresponding representative BURP genes.

### Characterization of *MtBURP* Proteins

Information regarding the MtBURP sequences, such as the number of amino acids, molecular weights (MW) and PIs, was obtained using ProtParam^3^. Multiple sequence alignment of the *MtBURP* proteins was performed with Clustal X and calculated using MEGA v4.0. Determinations of the signal peptides were performed with SignalP^4^.

### Motif Distribution and Exon-Intron Structure of *MtBURP* Proteins

The conserved motifs encoded by each *MtBURP* gene were investigated. Protein sequences were subjected to online Multiple Expectation Maximization for Motif Elicitation (MEME^5^). Parameters were set as follows: the occurrence of a single motif was zero or one per sequence; minimum motif width was 10; maximum motif width was 200 and maximum number of motifs to find was 15; all other parameters were default values. To predict the exon-intron structure of the *MtBURP* genes, comparison of the genomic sequences and their predicted coding sequences (CDS) was performed using GSDS^6^ (Guo et al., 2007). To analyze whether the motifs in each putative BURP gene was implicated in stress responses, 2,000-bp genomic sequences upstream of the transcription start coden (ATG) were acquired from (www.phytozome.net/search.php). PLACE^7^, an online database of plant *cis*-acting regulatory DNA elements (*cis*-element) (Higo et al., 1999), was employed to investigate putative *cis*-elements in the promoter regions of the *MtBURP* genes.

### Calculation of Ka/Ks Values

The synonymous (Ks) and non-synonymous (Ka) nucleotide sub-stitution rates and the Ka/Ks values were calculated for each pair of duplicated *Medicago* BURP proteins. Protein sequences of the gene pairs were aligned using Clustal X (Thompson et al., 1997), and the results were used to guide the codon alignments by PAL2NAL^8^ (Stacey et al., 2006). The generated codon alignments were subjected to computation of Ks and divergence levels (Ka/Ks ratios) using DnaSP software (version 5.10), and these values were then translated into divergence time in millions of years assuming a rate of 6.1 × 10^-9^ substitutions per site per year. The divergence time (T) was calculated as T = Ks/2λ (λ = 6.1 × 10^-9^) (Juretic et al., 2005). A sliding window analysis of Ka/Ks ratios was performed with the following parameters: window size, 150 bp; step size, 9 bp.

### Microarray Analysis

The genome-wide microarray data were obtained from the *M. truncatula* Gene Expression Atlas (MtGEA) Project^9^. The expression data were gene-wise normalized. Hierarchical clustering analysis was conducted using clustering distance “correlation” (Pearson correlation) and the clustering method used “complete” (complete linkage method) in R (Robin et al., 2011). A heat map was generated in R using the pheatmap function (Robin et al., 2011).

### Plant Material and Treatments

The plant material used in this analysis were young *M. truncatula* seedlings, which were grown in a growth chamber with a continuous 30°C temperature, a photoperiod of 12 h/12 h, 80 μmolm^-2^s^-1^ photon flux density and 50% relative humidity were used. For drought stress, the seedling leaves were treated with 20% PEG-6000 (polyethylene glycol). Leaves of the stress-treated plants were collected at time intervals of 0, 2, 4, 8, 12, and 24 h. Control seedlings were not subjected to stress treatment (Zhou et al., 2012, 2014). After harvest, the materials were immediately frozen in liquid nitrogen and stored at -80°C for further analysis.

### Expression Analysis

Total RNAs of all the collected samples were extracted using the Trizol reagent (Bio Basic Inc, Canada). Subsequently, the RNAs were treated with DNase I (Promega, Madison, WI, USA) to remove contaminating genomic DNA. Approximately 1 μg of each DNase-treated RNA samples was reverse transcribed by Prime Script RT Master Mix (Takara, Dalian, China) at 37°C for 20 min in a 20 μl reaction sample. The amplification reactions were carried out using an SYBR Premix Ex Taq^TM^ II (Perfect Real Time) (Takara, Dalian, China) according to manufacturer’s instructions with three replicates (Zhou et al., 2012, 2014). The specific primers of each BURP gene (**Supplementary Table S1**) were designed using Primer5.0, and their specificity was checked using information provided on the NCBI website. SEC (TC77416) (Kuppusamy et al., 2004), a constitutively expressed *Medicago* housekeeping gene, was used as reference for normalization. The PCR conditions were as follows: 95°C for 10 min, 40 cycles of 15 s at 95°C, 58°C for 1 min. Three technical replicates were taken in each biological replicate. The relative expression level was calculated as 2^-ΔΔCT^ [ΔC_T_ = C_T,Target_ – C_T,CY P2_. ΔΔC_T_ = ΔC_T_, _treatment_ – ΔC_T,CK(0__h)_]. The relative expression level (2^-ΔΔCT,CK(0h)^) in the control plants without treatment was normalized to 1 as described previously. Statistical analysis was performed using SDS software 1.3.1 (Applied Biosystems).

## Results

### Identification and Annotation of BURP Genes in *Medicago truncatula*

To identify all putative genes of BURP family in the *Medicago* genome, the HMM profiles of the BURP domain were employed as a query to search against the protein database of *M. truncatula* using the BlastP program. Through this approach, a total of 43 BURP domain-containing sequences were identified. A complete sequence alignment in MEGA 4.0 was used to identify 39 non-redundant BURP genes in *M. truncatula*. These 39 *MtBURPs* were entered into the Pfam and SMART databases to ensure that they all contain the BURP domain. The total number of BURP genes identified in *M. truncatula* (39) is greater than that in other species, such as poplar (18) (Shao et al., 2011), rice (17) (Ding et al., 2009), soybean (23) (Xu et al., 2010) and sorghum (11) (Gan et al., 2011). Information about the putative *MtBURP* proteins, including the number of amino acids (length), MW, isoelectric point (PI) and physical location on the chromosomes, is listed in **Table 1**. The lengths of the *MtBURP* protein sequences range from 87 aa (*MtBURP38*) to 630 aa (*MtBURP33*), while the MWs of these *MtBURP* proteins range from 9.6231 kDa (*MtBURP38*) to 70.6707 kDa (*MtBURP26*). The lowest PI of the *MtBURP* proteins is 4.55 (*MtBURP32*), while the highest is 9.62 (*MtBURP26*).

**Table 1:** Sequence characteristics of 39 BURP genes identified in *Medicago truncatula*.

Gene name	Sequence ID	Deduced polypeptide			Chr	Chr. Locations	Signal P position	Cleavage Site
		Length (aa)	MW (kDa)	PI				
MtBURP1	Medtr1g007520.1	325	36.0253	8.7	1	0.989854	1–21	TNA-TM
MtBURP2	Medtr1g008420.1	217	24.5913	9.33	1	1.470671	1–21	THA-AL
MtBURP3	Medtr1g008450.1	313	34.7706	6.83	1	1.478971	1–21	THA-TL
MtBURP4	Medtr1g008460.1	305	34.2124	8.67	1	1.48444	No	
MtBURP5	Medtr1g008470.1	325	36.8425	8.06	1	1.491672	No	
MtBURP6	Medtr1g008500.1	385	41.4282	8.87	1	1.504798	1–21	THA-AL
MtBURP7	Medtr1g008510.1	326	36.5821	7.63	1	1.511292	1–21	TNA-AI
MtBURP8	Medtr1g008530.1	327	36.8692	6.86	1	1.517723	1–21	TNA-EI
MtBURP9	Medtr1g008560.1	330	37.0536	7.16	1	1.529702	1–21	TNA-AM
MtBURP10	Medtr1g008580.1	323	36.2235	6.64	1	1.537707	1–21	TNA-TV
MtBURP11	Medtr1g008590.1	325	36.0043	8.7	1	1.544253	1–21	TNA-VM
MtBURP12	Medtr1g045860.1	269	29.9221	6.45	1	13.74125	1–21	ISA-TT
MtBURP13	Medtr2g081590.1	273	30.5491	6.26	2	24.542149	1–23	SHA-SV
MtBURP14	Medtr2g081610.1	268	29.81	5.73	2	24.549179	No	
MtBURP15	Medtr2g081770.1	278	31.0876	5.52	2	24.61816	1–23	AFA-GE
MtBURP16	Medtr3g116380.1	307	34.7511	6.21	3	1.981704	1–26	SCA-RK
MtBURP17	Medtr3g078090.1	628	68.6082	9.41	3	24.944282	1–20	SIA-TI
MtBURP18	Medtr3g107830.1	264	30.0483	8.1	3	38.355243	No	
MtBURP19	Medtr3g109490.1	322	35.6941	8.11	3	39.318334	1–21	THA-AL
MtBURP20	Medtr3g116270.1	512	58.8735	6.63	3	41.93637	1–25	GYA-RD
MtBURP21	Medtr3g116320.1	565	64.3098	6.63	3	41.951485	1–24	ASC-AR
MtBURP22	Medtr3g116410.1	285	32.3102	6.7	3	42.003836	1–26	DQA-RE
MtBURP23	Medtr4g068620.1	128	14.3274	6.12	4	21.664271	No	
MtBURP24	Medtr4g069680.1	296	33.4937	8.16	4	21.786799	1–26	SRA-RE
MtBURP25	Medtr4g133840.1	190	20.9981	6.99	4	47.42306	No	
MtBURP26	Medtr5g033330.1	621	70.6707	9.62	5	13.95499	No	
MtBURP27	Medtr5g033360.1	225	25.8113	9.22	5	13.960123	16-Jan	CDG-RK
MtBURP28	Medtr5g033410.1	406	46.3597	5.98	5	13.97349	No	
MtBURP29	Medtr5g033420.1	226	27.0057	6.92	5	13.975735	No	
MtBURP30	Medtr5g033430.1	121	14.4494	5.93	5	13.977282	No	
MtBURP31	Medtr5g033440.1	200	23.6538	4.95	5	13.979471	No	
MtBURP32	Medtr5g033450.1	305	33.8686	4.55	5	13.981007	1–22	CFA-QE
MtBURP33	Medtr5g034320.1	630	68.7653	8.57	5	14.438111	1–23	ISA-GD
MtBURP34	Medtr6g086530.1	209	23.3262	8.84	6	20.258771	No	
MtBURP35	Medtr8g045890.2	523	59.6125	5.95	8	11.990199	1–24	SKS-GE
MtBURP36	Medtr8g045970.1	452	51.7236	5.68	8	12.020602	No	
MtBURP37	Medtr8g046000.1	236	25.7667	6.59	8	12.033004	1–17	ALA-GI
MtBURP38	Medtr8g075930.1	87	9.6231	6.94	8	20.582426	No	
MtBURP39	AC235667_9.1	325	37.2305	7.07	0	20.440642	1–21	INA-SQ

### Chromosomal Locations of *MtBURP* Genes

**FIGURE 1:**
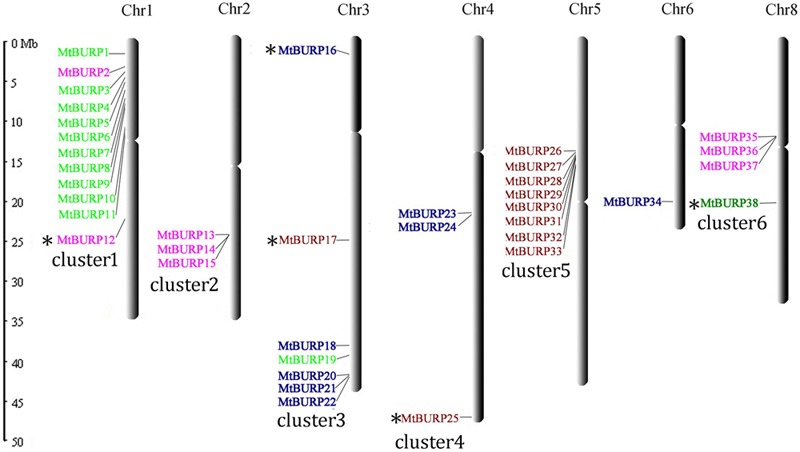
**Physical locations of *MtBURP* genes on *Medicago truncatula* chromosomes.** Gene names in different subfamilies are highlighted with various colors. And these colors were corresponding to the results in **Figure 2**, which represent different subfamilies. All BURP genes distributed in different chromosomes belong to cluster1–6 except chromosome 6. The “^∗^”represent that these genes were no clustered together with each other.

Thirty-eight out of 39 BURP genes are distributed unevenly across all of the chromosomes of *M. truncatula* except for chromosome 7,while only one gene (*BURP39*) was mapped to as yet unattributed scaffolds (**Figure 1**). The number of *MtBURP* genes on each chromosome varies widely. The largest number of BURP genes was found on chromosome 1 (12 genes), followed by chromosome 5 (eight genes). By contrast, chromosome 6 contains only one BURP gene. Three genes are located on chromosomes 2, and three genes are located on chromosome 4. Seven genes are distributed on chromosome 3. Four genes were identified on chromosomes 8. These results show that the distribution pattern of each type of BURP gene is highly irregular. The authors also noticed that BURP genes on chromosome 2 and 5 clustered together with each other. The BURP genes on chromosomes 1, 3, 4, and 8 clustered together with each other except *MtBURP12, MtBURP16, MtBURP17, MtBURP25*, and *MtBURP38*. The names of the BURP genes (from *MtBURP1* to *MtBURP39*) were determined according to their positions on the chromosomes. This nomenclature system provides a unique identifier for each member in a gene family. Such a system has previously been used in genome-wide analysis, including studies of the auxin response factor (ARF) gene in *Arabidopsis* (Nakano et al., 2006) and rice (Wang et al., 2007).

In *M. truncatula*, the 38 BURP genes are present in six clusters. According to Holub (2001), a gene cluster is a chromosomal region containing two or more genes within a 200-kb region. Each *M. truncatula* chromosome contains a BURP gene cluster, except for chromosome 6. A high-density region was identified, containing ten BURP genes (*MtBURP2*–*11*) on a 0.6 Mb region of chromosome 1; the authors therefore determined that region, is a hot spot of BURP gene distribution.

### Phylogenetic Analysis of *MtBURPs*

**FIGURE 2:**
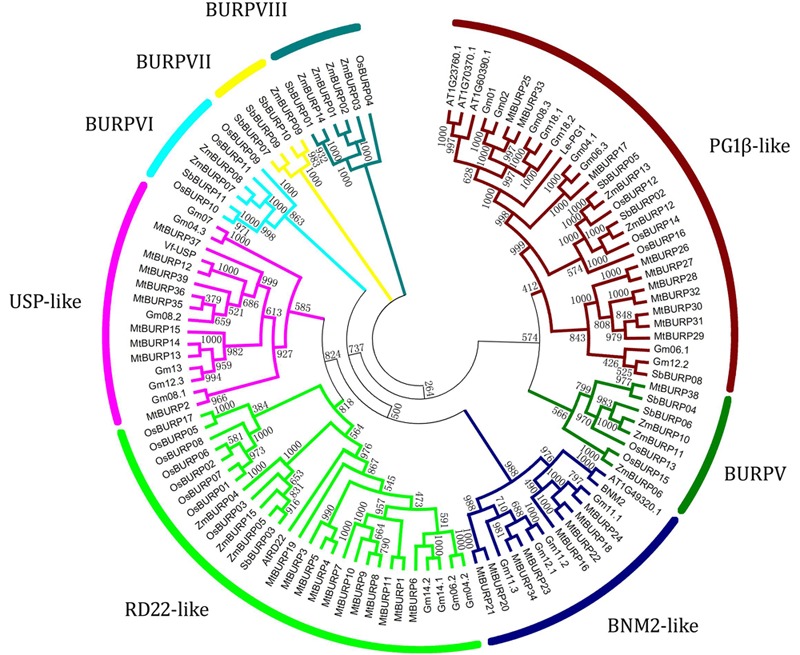
**Phylogenetic tree of *Medicago truncatula, Arabidopsis, Lycopersicon*, and *Vicia* BURP genes.** The unrooted tree was generated using the Clustal X program with the neighbor-joining method. The bootstrap values are indicated on the branches. At, *Arabidopsis thaliana*; Le, *Solanum lycopersicum*; Mt, *Medicago truncatula*; Vf, *Vicia faba*.

The abundance of *MtBURP* genes, compared to that in other plant species, may be derived from multiple gene duplication events, represented by a whole-genome duplication following multiple segmental and tandem duplications. To verify this hypothesis, a phylogenetic tree was constructed from the 113 BURP sequences, including 17 rice, 11 sorghum, 15 maize, 23 soybean, 39 *Medicago*, 4 BURP proteins in *Arabidopsis* (*AT1G49320.1, AT1G70370.1, AT1G23760* and *AT1G60390.1*) and 4 host BURP members (*AtRD22, Vf-USPs, BNM2* and *Le-PG1β*) (Gan et al., 2011). Based on the domain composition and phylogenetic relationship of the protein sequences, the 113 plant BURP proteins could be divided into eight subfamilies: BNM2-like, RD22-like, USP-like, PG1β*-*like, BURPV, BURPVI, BURPVII, BURPVIII (Ding et al., 2009). Interestingly, all the members in the BURPVI and BURPVIII subfamilies are exclusively from rice, maize and sorghum, belonging to monocotyledon, which suggests that the main characteristics of this family in plants were generated before the dicot-monocot split. Likewise, the members of the BNM2-like subfamily and USP-like subfamily were all from dicotyledons, while RD22-like subfamily, BURPV subfamily and PG1β*-*like family contained members from both dicotyledon and monocotyledon species. Interestingly, BURPVII subfamily contained members from maize and sorghum that belong to the same family of plants. This suggested that the functions and evolution of the BURP genes might have some variation in dicotyledon and monocotyledon (**Figure 2**).

**FIGURE 3:**
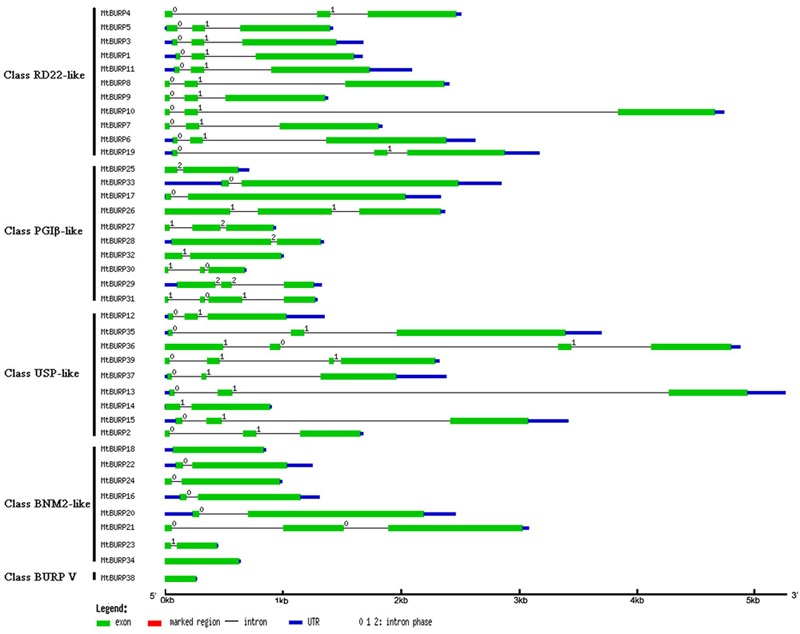
**The structure of exon-introns in *MtBURP* genes.** Exons, intron and UTRs are indicated with different colored lines. The length of each *MtBURP* gene can be estimated using the scale at the bottom of the figure.

The exon-intron structural analysis of the *MtBURP* genes was also performed by the authors. Each CDS of the *MtBURP* genes is disrupted by one or more exons (**Figure 3**), indicating that the BURP protein diversity. The number of exons in each gene varies widely among the *MtBURP* genes. The largest number is 4, such as *MtBURP31, -36* and *-39*. By contrast, the *MtBURP38, -18* and *-34* contain only one exon.

In preliminary analysis, gene duplication and divergence events were found to be the main contributors to evolutionary momentum. A gene duplication event is commonly defined by the following criteria (Gu et al., 1998; Yang et al., 2008): (1) the alignment sequence covers ≥70% of the longer gene; (2) the similarity of the aligned regions ≥70%. Among the 39 *MtBURP* genes, the authors found that six gene pairs (*MtBURP4/Mt BURP5, MtBURP1*/*MtBURP11, MtBURP28*/*MtBURP32, MtBUR P13*/*MtBURP14, MtBURP18*/*MtBURP22*, and *MtBURP20/MtB URP21*) participated in gene duplication events.

### Strong Purifying Selection for BURP Genes in *Medicago*

Assuming that synonymous silent substitutions per site (Ks) occur at a constant rate over time, the dates of the large-scale duplication events can be estimated (Maher et al., 2006). To explore the divergence fate of different duplicated BURP genes, the Ka (non-synonymous substitution rate) and the Ks (syn-onymous substitution rate) and the Ka/Ks ratios were calculated for each duplicated *MtBURP* gene pair. Generally, Ka/Ks ratios <1 are indicative of functional constraints with negative or purifying selection of the genes; a ratio = 1 indicates neutral selection and ratios >1 indicates accelerated evolution with positive selection (Juretic et al., 2005). Our study showed that the Ka/Ks ratios from 6 *Medicago* BURP duplicated gene pairs (**Table 2**) were less than 0.6 except *MtBURP4/MtBURP5*. This result suggests that the *Medicago* BURP gene family has evolved mainly under the influence of strong purifying selection pressure, with limited functional divergence occurring after duplication, and the BURP genes are slowly evolving at the protein level. Based on the divergence rate of 6.1 × 10^-9^ synonymous mutations per synonymous site per year as previously proposed for Legumes, the duplication that gave rise to the six duplication gene pairs were estimated to have occurred between 5.38 and 31.77 million years ago (Mya) (**Table 2**).

**Table 2:** Divergence between paralogous BURP gene pairs in *Medicago*.

Group	No.	Paralogous pairs	Ka	Ks	Ka/Ks	Duplication date (MY)	Duplicate Type
RD22	1	MtBURP1-MtBURP11	0.017	0.066	0.2599	5.385245902	T
	2	MtBURP4-MtBURP5	0.084	0.136	0.6166	11.13114754	T
BNM2	4	MtBURP18-MtBURP22	0.101	0.296	0.3414	24.2704918	T
	5	MtBURP20-MtBURP21	0.124	0.346	0.3578	28.3442623	T
PG1β	9	MtBURP28-MtBURP32	0.042	0.08	0.5267	6.581967213	T
USP	12	MtBURP13-MtBURP14	0.109	0.388	0.2804	31.7704918	T

**FIGURE 4:**
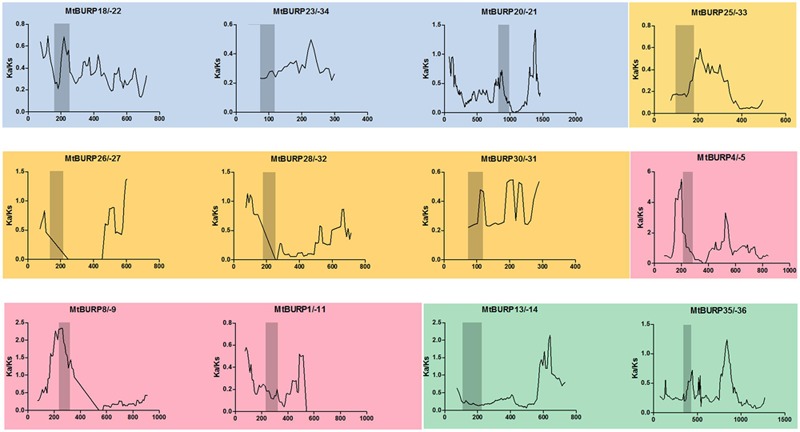
**Sliding window analysis employed to estimate selective pressures on paralogous BURP gene pairs in *Medicago*.** Four different colors reprent that paralogous BURP gene pairs came from different subfamilyes. As shown in the key, the gray blocks, indicate the positions of the BURP domain. The window size is 150 bp, and the step size is 9 bp.

To better understand the evolutionary constraints acting on this gene family, we compared all Ka/Ks values for 12 unambiguous pairs of BURP paralogs (including six duplicated gene pairs), since positive selection at a few individual codon sites can be masked by overall strong purifying selection, we performed a sliding-window analysis of Ka/Ks between each pair of BURP paralogs. As expected from the basic Ka/Ks analysis, sliding window analysis clearly showed that numerous sites/regions are under moderate to strong negative selection (**Figure 4**). As shown in **Figure 4**, the conserved domains of BURP, described as VXCHXCHX23-27CHX23-26CHX8W regions, are mainly subjected to strong purifying selection, with Ka/Ks ratios <1. Moreover, the domains of BURPs generally had lower Ka/Ka ratios (valleys) than the regions outside of them (peaks). There were a few exceptions to the generally low Ka/Ka ratios in domains. For instance, the gene pairs *MtBURP4/-5* and *MtBURP8/-9*, revealed sites with Ka/Ka ratios >1 in the BURP domain, indicating positive selection in this region. Divergence fate was also found between only paralogous pairs and duplicated gene pairs. While they all have the common characteristic, the Ka/Ks ratios of the conserved domains of BURP in duplicated gene pairs were all lower than paralogous pairs in the same subfamily. For example, *MtBURP14/-13* with ka/ks <0.5, while the same subfamily gene pairs, *MtBURP35/-36*, the Ka/Ks value >0.5.

### Sequence Analysis of *MtBURP* Genes

**FIGURE 5:**
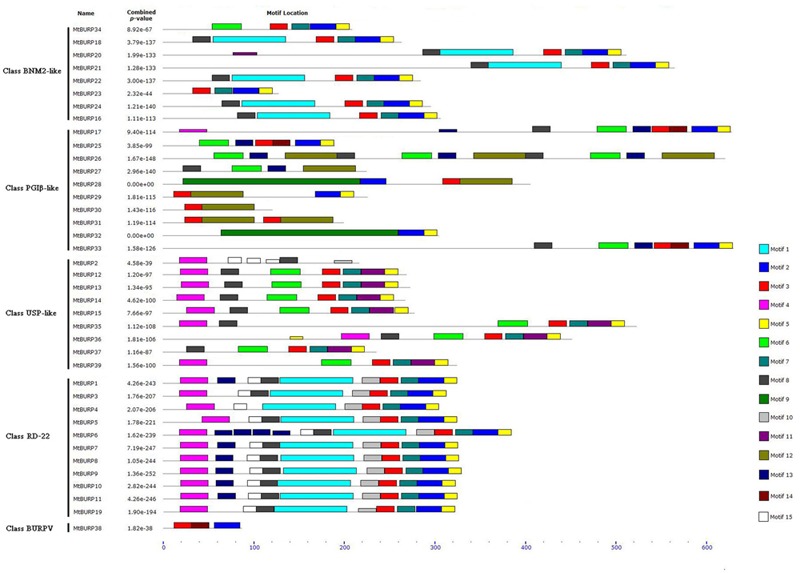
**The 15 putative motifs identified in the *Medicago truncatula BURPs* by multiple expectation maximization for motif elicitation (MEME).** Different motifs are indicated by different colors and shown on the right. Names of all members and *P*-value are shown on the left. Among the 15 conserved motifs, nine are BURP motifs (i.e., motif 1, 2, 3, 5, 6, 8, 9, 11, and 12). Other motifs with unknown functions were also found; perhaps these motifs are required for specific protein functions.

**FIGURE 6:**
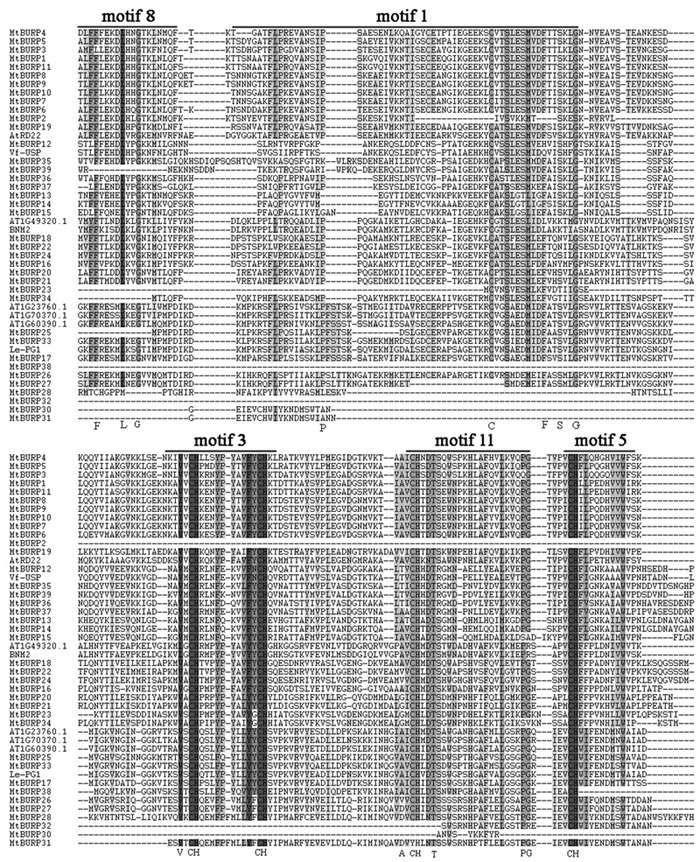
**Multiple alignments of *MtBURP* domain-containing proteins.** The complete amino acid sequences of 39 *MtBURP* proteins and eight representative BURP proteins were aligned using Clustal X. Motif (n) represents the motifs identified in *Medicago truncatula* BURP proteins by MEME. Conserved domains are shaded in black.

To determine whether the *MtBURP* proteins contain signal peptides, the putative *MtBURP* protein sequences were searched in the SMART database. Twenty-five *MtBURP* proteins contain signal peptides, and their predicted cleavage sites vary. Fifteen conserved motifs (**Figure 5**) were identified in the *MtBURP* proteins using the MEME web server. Each putative motif obtained from MEME was annotated by searching the Pfam and SMART databases. Among the fifteen conserved motifs that were identified, nine are BURP motifs (i.e., motif 1, 2, 3, 5, 6, 8, 9, 11, and 12). Other motifs with unknown functions were also found; perhaps these motifs are required for specific protein functions. Detailed information about conserved amino acid sequences and lengths of the 15 motifs are shown in **Table 3**. Fifteen motif logo were showed as **Supplementary Figure S1**. Multiple protein sequence alignments were also performed with Clustal X (**Figure 6**). The alignment revealed the existence of several highly conserved amino acid residues, including two phenylalanines, one leucine, three glycines, two prolines, one cysteine, one serine, one valine, one threonine, one alanine and four CH motifs. Some proteins have low homology to the others, such as *MtBURP30* and *-32*. *MtBURP30* contains no amino acid residues. *MtBURP32*, has just one proline and one CH motif. In summary, the C-terminus of the *MtBURP* proteins can be described as VXCHX_11_CHX_25_AXCHX_2_TX_16_PGX_7_CH.

**Table 3:** Multiple expectation maximization for motif elicitation (MEME) motif sequences in predicted *MtBURP* proteins.

Motif	Width	Conserved amino acid sequences
1	80	QRTPRNGAKFWPREVANSIPFSSNKLENILNYFSIKQGSPEAEIMKRTISECEANPIKGEEKLCVTSLESMVDFTTSKLG
2	28	AVCHLDTSQWNPNHPAFQVLGVQPGTIP
3	19	NKWVMCHRENYPYAVFYCH
4	30	THAMLPPELYWKSMWPNTPMPKAITDLLHP
5	14	VCHWIPQDHMVWVP
6	32	CCERPPARGETKYCATSLESMIDFAISKLGRN
7	19	TRVYMVPLEGVDGTRVKAI
8	19	NVALFFREKDLHHGTKMNM
9	195	GSPNGEHRYEFGPGGQEKYADGEDSPNGEHKYEFGPSAQEKYTDDEGSPNGEHKYEFGPGAQENYTDGEGSPNGE HKYEFGPGTQDKYTDGKGSPYGEHSTNSDLAVPMGILLLSALSEFVLMFPIRTAFAIDILLLSARSEFVLMFPIRTAFAIGILH QEMFPFMLLYFCHYIPMGRFYEVEILDLQKIMINQA
10	19	NESGLQQYVIAKGVKKLGE
11	26	VCHHDTRGMNPDMLYEILKVDPGTIP
12	57	HYIPMARFYEVEVLDLQKIMINQAVDVCHLNTSSWSRNHPTFLELGSTPGEIEWCHW
13	19	VDGSKGGVMVGRRKGYEGG
14	19	YIPMVRVYQVEILDLQRIM
15	14	YNYAASETQLHDKQ

### Identification of Putative Stress-Related *cis*-Elements in the Promoters of the *MtBURP* Genes

**FIGURE 7:**
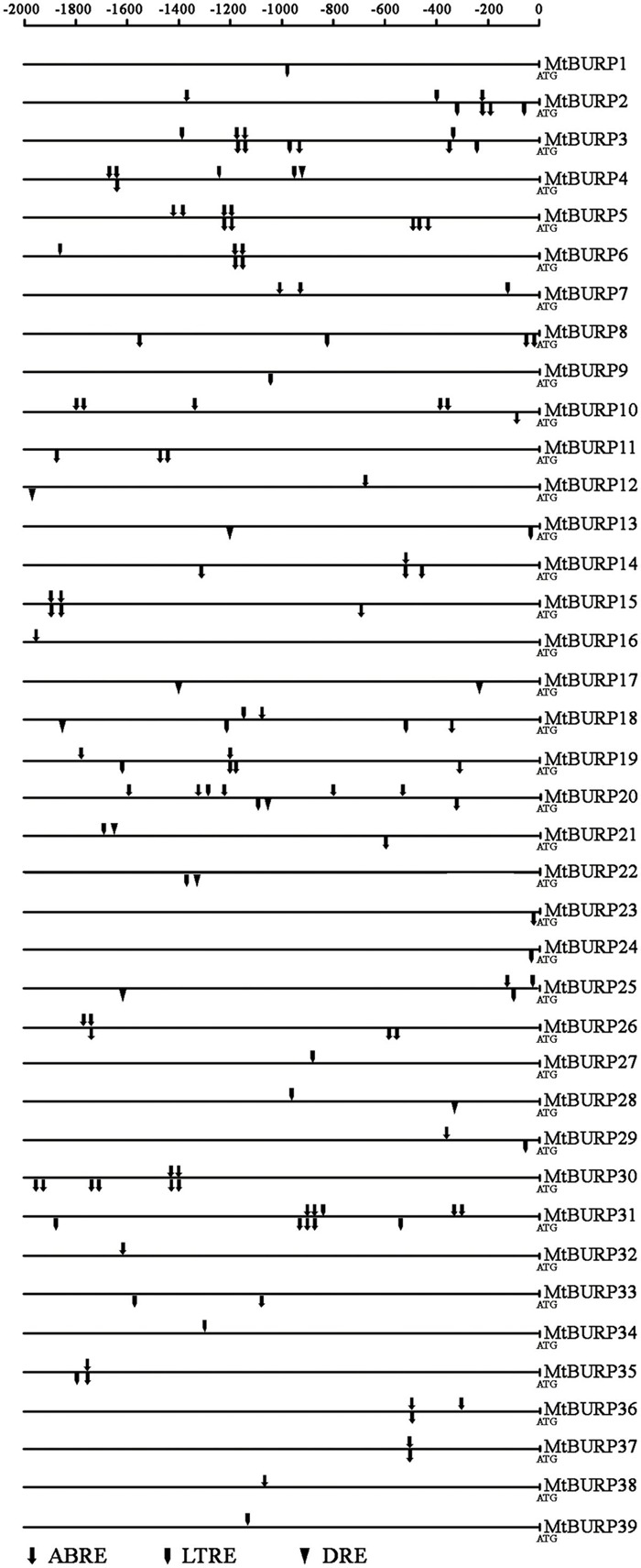
**Distribution of major stress-related *cis*-elements in the promoter sequences of *MtBURP* genes.** Putative ABRE, LTRE and DRE core sequences are represented by different symbols as indicated. The *cis*-elements distributed on the sense strand and reverse strand are indicated above and below the black lines, respectively.

To elucidate the possible regulatory functions of *MtBURP* genes in various stress responses, the authors identified putative stress-related *cis*-elements in the 2-kb promoter regions upstream of the transcription start coden (ATG) of stress-responsive *MtBURP* genes (**Figure 7**). Three types of *cis*-elements, which are directly related to the low temperature-responsive element (LTRE) (Yamaguchi-Shinozaki and Shinozaki, 2005), ABA-responsive element (ABRE) (Narusaka et al., 2003; Yamaguchi-Shinozaki and Shinozaki, 2005) or dehydration-responsive element (DRE) (Narusaka et al., 2003), were detected by searching the promoter sequences against the PLACE database. All *MtBURP* genes from each group were analyzed. Surprisingly, the authors found that 39 *MtBURP* genes contained at least one putative ABRE, LTRE or DRE in their promoter regions. Moreover, some members contain multiple ABREs, LTREs and DREs in their promoter regions, such as *MtBURP2, -6* and *-19*. In addition, some members, such as *MtBURP16* and -*32*, have only one putative stress-related *cis*-element. Among the stress-related *cis*-elements, ABREs are more common than LTRE or DRE, which indicates that *MtBURP* genes may play an important role in regulating the level of ABA in the plant. As a few BURP family genes (such as RD22) have been reported for their responsiveness to drought stresses, the transcript levels of all the *MtBURP* genes were investigated in leaves under drought treatment in this study to obtain a complete view of the stress responsiveness in the *Medicago* BURP family.

### Microarray Analysis

**FIGURE 8:**
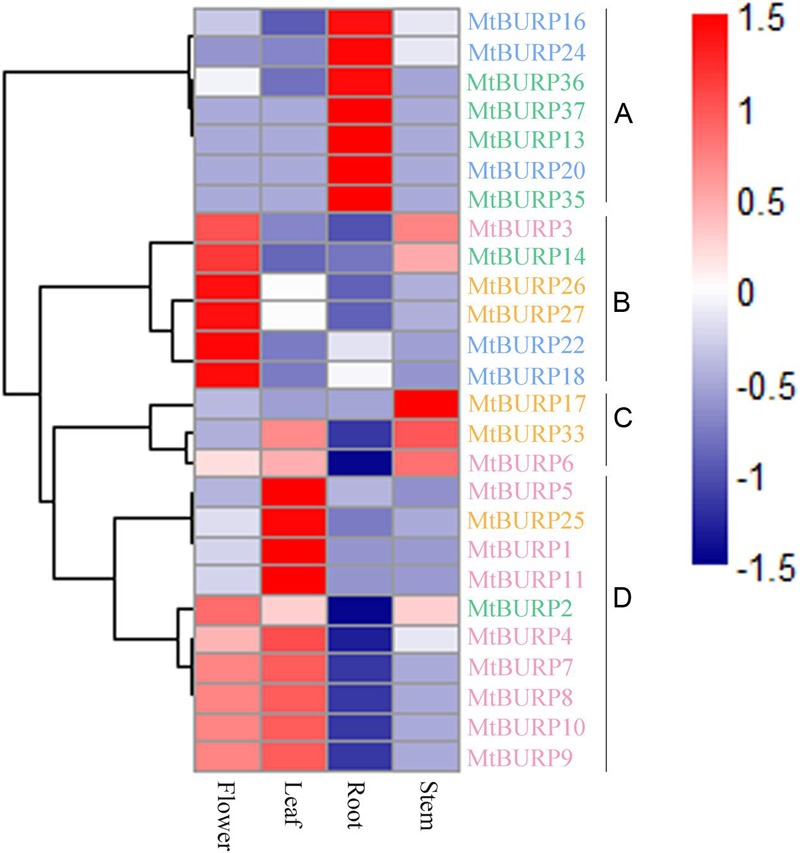
**Hierarchical clustering of *MtBURP* proteins in four tissues of *Medicago truncatula*.** RNA-seq relative expression data from 4 tissues were used to reconstruct the expression patterns of *Medicago* genes. The raw data was normalized and retrieved from the online database (http://mtgea.noble.org/v2/). Gene names in different subfamilies are highlighted with various colors. Genes clustered into four groups (A–D) are indicated by the black vertical bars.

To gain insight into the expression patterns of *Medicago* BURP genes in various tissues, we searched the Gene Expression Atlas (MtGEA) of *Medicago*, this atlas provides high-resolution gene expression data from 4 diverse tissues, including aerial tissues (leaf, flower, stem), underground tissues (root). Because the expression profiles of 13 BURP genes (*MtBURP12, -15, -19, -21, -23, -28, -29, -30, -31, -32, -34, -38, -39*) weren’t obtained in the database, we only examined the expression patterns of 26 BURP genes (**Figure 8**). Most *Medicago* BURP genes exhibit broad expression patterns (**Figure 8**). 26 *Medicago* BURPs are expressed in all of the four tissues (leaves, flowers, roots, stems). The heat map also revealed that the majority of *MtBURPs* showed preferential expression. Based on a hierarchical clustering analysis, 26 BURP genes were mainly clustered into four groups (A–D) (**Figure 8**). Group A showed partial expression in roots, group B in flowers, group C in stems and group D in leaves. 15 *MtBURPs* (*MtBURP16, -24, -36, -37, -13, -20, -35, -26, -27, -22, -18, -17, -5, -1*, and *-11*) showed marked high transcript abundance profiles in only a single tissue. Among the 26 *Medicago* BURP genes examined, six showed the highest transcript accumulation in flowers (*MtBURP3, -14, -18, -22, -26*, and *-27*), 7 in roots (*MtBURP13, -16, -20, -24, -35, -36*, and *-37*), one in stems (*MtBURP17*) and four in leaves (*MtBURP1, -11, -5*, and *-25*; **Figure 7**). Genes in different subfamilies have their primary abundant transcripts, for instance, BNM-like in flowers and stems, USP-like in flowers, stems and roots, RD22 in leaves, flowers and stems and PG1 in stems, flowers and leaves (**Figure 8**). These subfamily specific tissue expression patterns may be closely related to gene functions. The expression patterns of the paralogous pairs were also revealed by heat maps; paralogous pairs with high sequence similarity have similar expression patterns. The best examples of this include *MtBURP18/-22, MtBURP26/-27, MtBURP35/-36* and *MtBURP8/-9*, which are strongly expressed in leaves, flowers and roots respectively, with little or no expression in other tissues. Expression divergence was also found in paralogous pairs. For example, *MtBURP14* is highly expressed in flowers, while its paralog, *MtBURP13*, is highly expressed in roots.

### Expression Levels of *Medicago* BURP Genes in Response to Drought Stress

**FIGURE 9:**
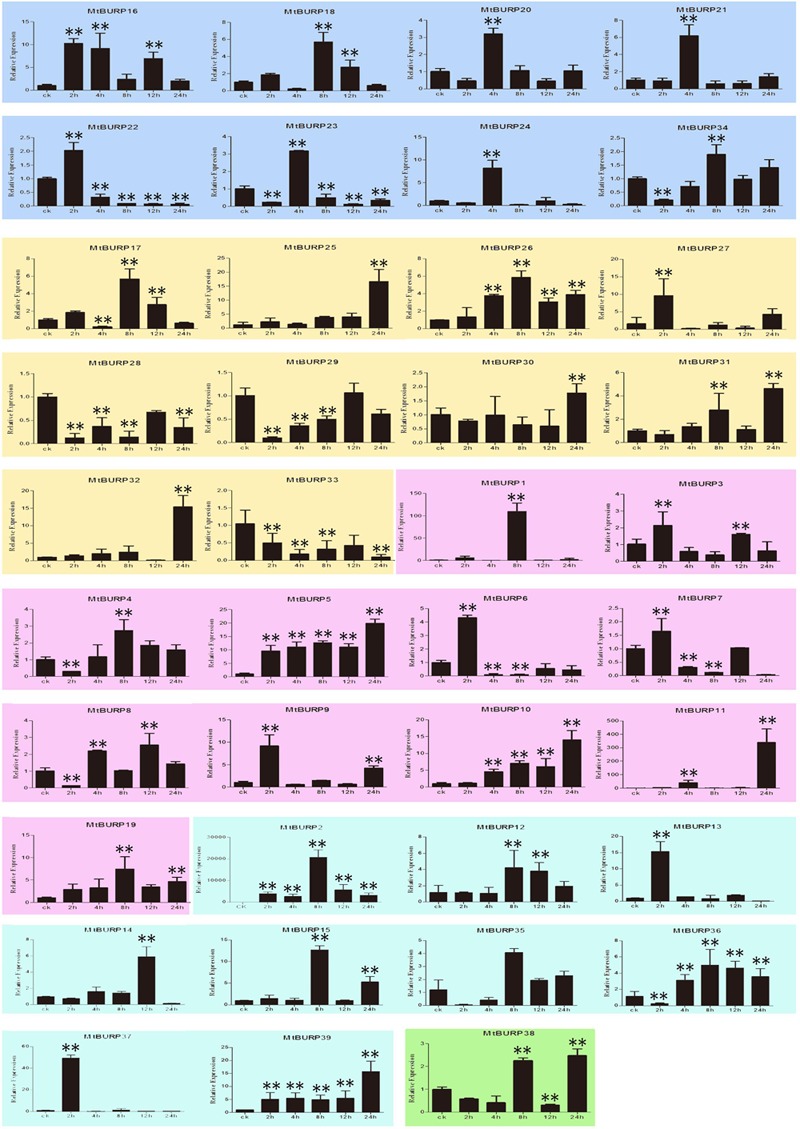
**Expression patterns of the 39 BURP genes under drought stress.** Relative expressions of BURP genes were examined by qRT-PCR and normalized by SEC expression. Different subfamilies were presented in different background color. The Y axis is the scale of the relative expression level. The X axis is the time course of drought stress treatment. Mean value and SDs were obtained from three biological and three technical replicates. Two asterisks (^∗∗^*P* < 0.01, Student’s *t*-test) represent significant differences between the controls and treatments.

The BURP genes have been reported to affect the plant development, as well as to respond and adapt to stresses. To examine the drought stress responsiveness of the BURP family genes in *Medicago*, the expression patterns of the BURP genes in 3-week-old leaves under drought stress, one of the serious environmental stresses affecting *Medicago* production, was investigated. As shown in **Figure 9**, the expression levels of all the 39 BURP genes corresponding to early (0–8 h) or late (12 and 24 h) response to drought stress were compared with normal plants, but some differences were observed among these genes. For instance, only two genes (*MtBURP33* and -*28)* both included in PG1*β* were down-regulated and 10 genes (*MtBURP10*, -*25*, -*26*, -*5*, -*16*, -*19*, -*39*, -*11*, -*15*, and -*2*) were up-regulated by drought treatment through all the time points. Among the 10 up-regulated genes, four genes (*MtBURP5*, -*10*, -*11*, and -*19*) belong to RD22-like, two genes (*MtBURP25* and -*26*) to PG1β, one genes (*MtBURP16*) to BNM2-like and others (*MtBURP2*, -*15* and *-39*) to USP-like. Interestingly, *MtBURP11* from RD22-like subfamily and *MtBURP15* from USP-like subfamily reach the maximum at certain time point respectively, which is different to other up-regulated genes. *MtBURP11* slowly up-regulates in the time range 2–12 h, and reaches the maximum at 24 h, while *MtBURP15* reaches the maximum at 8 h, with a slight up-regulation in other time ranges. Notably, the authors found that every subclass family at least had one gene strongly up-regulated by drought stress except subfamily BURPV. Interestingly, the differential expression patterns were also found among the same subfamily. Besides the above-mentioned 12 genes with regular up-regulation or down-regulation after drought treatment, other 27 genes also showed responses to drought treatment. As shown in **Figure 9**, it should be noted that *MtBURP31*, -*4*, -*36*, -*12*, -*35*, and -*8* were down-regulated at slight (0–8 h) drought stress but were dramatically up-regulated thereafter. Whereas *MtBURP22*, -*6*, -*23*, and -*37* were up-regulated at slight drought stress but down-regulated at moderate (12 h) and severe (24 h) drought treatment. The authors also pay more attention to these genes of *MtBURP32*, -*24*, -*20*, -*21*, -*14*, -*13*, -*1*, -*17*, -*27*, -*18*, and -*9* which showed major expression changes. They were expression peaked at one time point after drought treatment, and their relative expression were not all the same when they were expression peaked. The lowest is 4 (*MtBURP20*), while the highest is 150 (*MtBURP1*). Other time points showed minor changes might also have specific functions in *Medicago* under drought treatment. The highest expression levels of *MtBURP3* and -7 of RD22-like subfamily were found at 2 and 12 h after treatment, while those of *MtBURP34*, -*30*, -*29*, and -*38* were observed later, which listed as follow, *MtBURP38* and -34 were found at 8 and 24 h, *MtBURP30* at 24 h and *MtBURP29* at 12 h after treatment.

## Discussion

Preliminary analysis of BURP gene family has been performed in abundant plant as mentioned earlier. However, this family has not previously been studied in *Medicago*. In the present study, an overall analysis of the BURP gene family in *Medicago* was performed, including analysis of their phylogeny, chromosomal location, gene structure, conserved motifs and expression profiles. A total of 39 non-redundant BURP genes were identified in the *M. truncatula* genome, which is 1.7 times that of Soybean (Xu et al., 2010). This difference might be attributed to the fact that the identification of BURP genes in this study was based on the amino acid sequence using BlastP program, whereas the analysis in the previous report was based on the nucleotide sequence using TblastN searches. Moreover, more and more sequences had been assembled and introduced into the *Medicago* genome database, this might be another reason caused the different number of the BURP genes. The lengths of these sequences varied remarkablely, suggesting a highly degree of complexity within the *MtBURP* gene family.

Gene duplication and divergence events are the main processes that multiply genetic material during evolution and selection (Pickett and Meeks-Wagner, 1995). The location of BURP genes in the *Medicago* genome may provide insights into the evolution of this gene family. In *M. truncatula*, 72% of *MtBURP* genes (28 out of 39) from six gene clusters are distributed on *Medicago* chromosomes. These gene clusters may have been derived from common ancestors respectively and may have been formed through a series of gene duplications. In *M. truncatula*, six sister pairs, including twelve BURP genes, have been identified. Gene duplication plays a major role in genomic rearrangement and expansion. Gene duplication events include tandem and segmental duplication events (Xu et al., 2010). Two or more genes located on the same chromosome result from tandem duplication events, while gene duplication between different chromosomes is a segmental duplication event (Xu et al., 2010). Interestingly, for all six sister pairs of *MtBURP* genes, including *MtBURP4*/*MtBURP5, MtBURP1*/*MtBURP11, MtBURP28*/*MtBURP32, MtBURP13/MtBURP14, MtBURP18/ MtBURP22* and *MtBURP20*/*MtBURP21*, both genes in a pair are on the same chromosome. In addition, all six sister pairs have undergone tandem duplication; the putative proteins encoded by the genes share more than 80% similarity at the amino acid level. In our analysis, all duplicated BURP gene pairs were involved in tandem duplication events, but there were no segmentally duplicated gene pairs, indicating that tandem duplication rather than segmental duplication served as the most important driving force throughout the long period of *Medicago* BURP gene evolution.

The ratio of non-synonymous versus synonymous substitutions (Ka/Ks) is an indicator of history of selection acting on a gene or gene region (Cristina et al., 1996). The Ka/Ks ratios of the 12 paralogous pairs showed that these gene pairs were subjected to purifying selection. Furthermore, the relatively higher Ka/Ks ratios for the *MtBURP4/-5* and *MtBURP28/-32* gene pairs may suggest that they experienced rapid evolutionary diversification following duplication. The domains of BURPs generally had lower Ka/Ka ratios (valleys) than the regions outside of them (peaks) except the gene pairs *MtBURP4/-5* and *MtBURP8/-9*, the authors suggested that is consistent with functional constraint being dominant in these domains. By calculating the duplication dates for the duplication gene pairs, we concluded that all of the duplication events in the *MtBURP* gene family occurred between 5.38 and 31.77 Mya. BURP domain-containing proteins are specific to plants (Wang et al., 2011). While this gene family has been identified in many plants, the functions of these genes are often unclear. Previous studies have suggested that BURP genes may play important roles in plant development and metabolism, and some BURP domain-containing proteins are responsive to stress treatment. To elucidate the functions of BURP genes in *M. truncatula*, stress-related *cis*-element analysis of the promoters of the *MtBURP* genes was performed, as well as Quantitative real-time PCR (qRT-PCR) analysis of the all *MtBURP* genes. From the RT-PCR analysis, the authors determined that the expression profiles of the BURP family in *Medicago* were complicated, suggesting that these genes performed a variety of physiological functions to help the plant adapt to environment challenges. The results showed that expression levels of all 39 genes were induced by drought stress, even though the induction of some genes was slight. It is noteworthy that *MtBURP19*, -*5*, -*11*, and -*10* were significantly up-regulated under drought stress. The author concluded that they may play an essential role in response to drought stresses. This conclusion was supported by the close relationship of these genes (*MtBURP19*, -*5*, -*11*, and -*10*) to *AtRD22*, which was previously identified as stress-response gene (Yamaguchi-Shinozaki and Shinozaki, 1993). The other six genes of this subfamily with minor expression changes may also have specific functions in *Medicago* under drought conditions (Xu et al., 2013). As reported, AtRD22 transcript was largely restricted to the leaf, that of AtUSPL1 was more prevalent in the root (Harshavardhan et al., 2014). We infered this phenomenon was also exist in *Medicago*, as showed in **Figure 8**, three up-regulated genes (*MtBURP2*, -*15*, and -*39*) to USP-like, two genes (*MtBURP2, -15*) were just up-regulated obviously at 8 h. Two down-regulated genes, *MtBURP33* and *-28*, belong to PG1β-like subfamily may be play positive roles in response to other stress, such as abiotic condition and cold condition. Intriguingly, the results of qRT-PCR were not always consistent with those of the promoter sequence analysis. For example, the promoter analysis showed that *MtBURP2*, -5, -*10*, -*16*, -*19*, -*25*, and -*26* have no DRE elements detected in the promoter regions, but the real-time PCR results showed that all these were induced by drought treatment. This strongly indicated that some unidentified stress responsive *cis*-elements might play important roles in regulating the *Medicago* stress response. A comparison of amino acid sequences and regulatory characteristics of the *MtBURP* gene pairs suggests that divergence in regulation evolves more quickly than functional divergence (Li, 1983).

## Conclusion

*Medicago truncatula* is a forage grass and a valuable herbal medicine (Crane et al., 2006). This plant is also useful for soil and water erosion control. However, due to inclement weather, the production of *M. truncatula* has been declining. Therefore, numerous stress-responsive genes from various protein families have been studied and used in genetic engineering to improve the stress tolerances of economic plants. In this study, the authors identified and characterized BURP genes in *M. truncatul*a, this work should promote further studies of the functions of stress-induced BURP domain-containing genes in this model species.

## Author Contributions

Conceived and designed the experiments: YL, XC. Performed the experiments: YL, XC. Analyzed the data: YL, XC, ZC, RC, HZ. Contributed reagents/materials/analysis tools: YL, XC, ZC. Wrote the paper: YL, XC, ZC. Guided the experiment: YX.

## Conflict of Interest Statement

The authors declare that the research was conducted in the absence of any commercial or financial relationships that could be construed as a potential conflict of interest.

## Footnotes

1 http://pfam.Sanger.ac.uk/

2 http://www.plantbreeding.wur.nl/uk/software_mapinspect.html

3 http://web.expasy.org/protparam/

4 http://www.cbs.dtu.dk/services/SignalP/

5 http://meme.sdsc.edu/meme/cgi-bin/meme.cgi

6 http://gsds.cbi.pku.edu.cn/

7 http://www.dna.affrc.go.jp/PLACE/signalscan.html

8 http://www.bork.embl.de/pal2nal/

9 http://mtgea.noble.org/v2/

## Supplementary Material

FIGURE S1 **Fifteen motif logo were created by online MEME**.

TABLE S1 **A list of primer sequences of the 39 selected MtBURP genes for qRT-PCR analysis**.
