# Supplementary figures and images for: Identification and Expression Analysis of BURP Domain-Containing Genes in Medicago truncatula
Source: Front Plant Sci. 2016 Apr 13;7:485. doi: 10.3389/fpls.2016.00485 (PMC4829796; doi:10.3389/fpls.2016.00485)

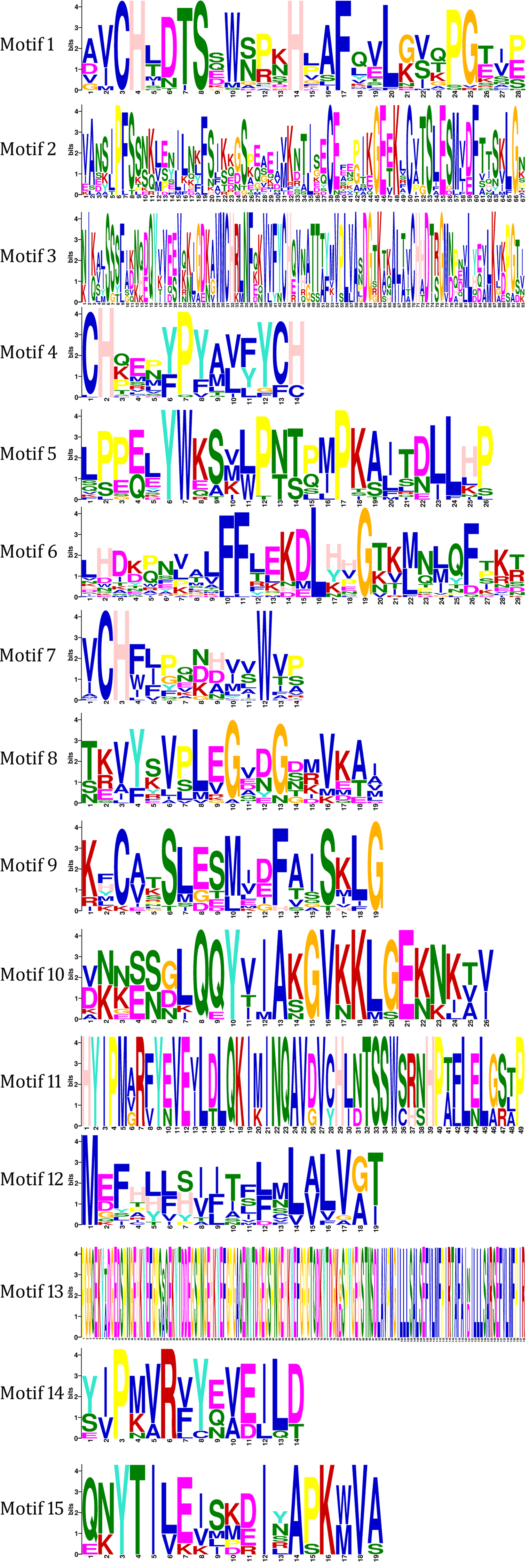

Supplement: FIGURE S1 — Fifteen motif logo were created by online MEME. [file Image_1.TIF]
