# Supplementary material for: Identification and Expression Analysis of BURP Domain-Containing Genes in Medicago truncatula
Source: Front Plant Sci. 2016 Apr 13;7:485. doi: 10.3389/fpls.2016.00485 (PMC4829796; doi:10.3389/fpls.2016.00485)
Supplement: TABLE S1 — A list of primer sequences of the 39 selected MtBURP genes for qRT-PCR analysis. [file Table_1.DOC]

**Table.S1**  A list of primer sequences of the 39 selected MtBURP genes for qRT-PCR analysis.

| Gene | Orientation | Primer Sequence(5’->3’) | Length | Tm | GC% | Product length |
| --- | --- | --- | --- | --- | --- | --- |
| BURP1 | Forward | TGC CAA AAG CCA TCA CTA ATC | 21 | 56.85 | 42.86 | 103 |
|  | Reverse | CTG ACT CCA ACA TCT ACA CCA CC | 23 | 60.62 | 52.17 |  |
| BURP2 | Forward | AAC AAC TCA AAT GCC AAA AGC C | 22 | 58.79 | 40.91 | 308 |
|  | Reverse | CCA AGA AGA ACA AGG CCA CAA | 21 | 58.97 | 47.62 |  |
| BURP3 | Forward | CTT TGG AAG GTG CTG ATG GA | 20 | 57.79 | 50 | 151 |
|  | Reverse | ACT TGA CCC TGT TGT AGG ATG TG | 23 | 60.24 | 47.83 |  |
| BURP4 | Forward | TTGCCTCGTGAAGTCGCT | 18 | 59.27 | 55.56 | 97 |
|  | Reverse | CAGATTCCGCTGATCCTTG | 19 | 55.79 | 52.63 |  |
| BURP5 | Forward | TATTTTCACTTACCATTTCGTTGC | 24 | 56.52 | 33.33 | 198 |
|  | Reverse | CACCCATGTTCCACCCTTTC | 20 | 58.74 | 55 |  |
| BURP6 | Forward | TTCGTCATTTATCCATCTTTATCG | 24 | 55.2 | 33.33 | 289 |
|  | Reverse | GCTGTTCCACCTGGCTTTGTT | 21 | 61.64 | 52.38 |  |
| BURP7 | Forward | GAAGTCCATGCTTCCCAACTC | 21 | 58.91 | 52.38 | 119 |
|  | Reverse | CATCTACACCACCCTTGCTTACA | 23 | 60.31 | 47.83 |  |
| BURP8 | Forward | CAACCGAAGTTTACTCTGTGCC | 22 | 59.78 | 50 | 169 |
|  | Reverse | TGATCCTGTGGGAGGATGTG | 20 | 58.79 | 55 |  |
| BURP9 | Forward | GCCAAAAGCCTTCACTAATCTG | 22 | 57.9 | 45.45 | 95 |
|  | Reverse | TGACTCCAACATCTAAACCACCA | 23 | 59.61 | 43.48 |  |
| BURP10 | Forward | AATGCTACAGTATCACCACCTCAA | 24 | 59.77 | 41.67 | 136 |
|  | Reverse | CATTATTGCTTACATCCCTTGCT | 23 | 57.22 | 39.13 |  |
| BURP11 | Forward | TCACTAATCTGCTACACCCTGCTG | 24 | 62.19 | 50 | 393 |
|  | Reverse | CAATCTGAACACCCTTTGACCCT | 23 | 60.75 | 47.83 |  |
| BURP12 | Forward | GAACGCAAACATTGTGTATCATCC | 24 | 58.6 | 41.7 | 258 |
|  | Reverse | TTTACCTCCATCGGGGGCTA | 20 | 59.8 | 55 |  |
| BURP13 | Forward | TGCTAGAACTTCTAAAGCCTGGAC | 24 | 60.32 | 45.83 | 165 |
|  | Reverse | CATAAGGTTGTGCTAAGGGACG | 22 | 59.06 | 50 |  |
| BURP14 | Forward | AACACTCCCATTCCCTCTTCTC | 22 | 59.43 | 50 | 194 |
|  | Reverse | TAAACGCCGTAAGGTTGTGC | 20 | 59.13 | 50 |  |
| BURP15 | Forward | TGGTCTTATCAAGTATCTTGGTGC | 24 | 58.57 | 41.67 | 110 |
|  | Reverse | TCTGTTCTCCTATGGTGGCTCT | 22 | 60.29 | 50 |  |
| BURP16 | Forward | TTTGAACCTATGGAAGGAGAGACA | 24 | 58.6 | 41.7 | 372 |
|  | Reverse | ATTAAGCACACGAAAGGCGACA | 22 | 58.2 | 45.5 |  |
| BURP17 | Forward | GAGGGAACCGAGTGTGGGAGAA | 22 | 63.8 | 59.1 | 320 |
|  | Reverse | TGTGACAGATGGCAACACCGTG | 22 | 61.9 | 54.5 |  |
| BURP18 | Forward | TGTGCCTTACCCTTATGCTGTT | 22 | 59.96 | 45.45 | 144 |
|  | Reverse | CTTGGTGCCCATTGTGATGT | 20 | 58.74 | 50 |  |
| BURP19 | Forward | GGTCCACACTCACCTTTTGCT | 21 | 60.75 | 52.38 | 295 |
|  | Reverse | GAATGGCTGCGTCTTCACAC | 20 | 59.83 | 55 |  |
| BURP20 | Forward | TTTGTTCACAGCGTTCTCGG | 20 | 59.07 | 50 | 240 |
|  | Reverse | CATGTCTCCATATTGACCCCTTA | 23 | 57.43 | 43.48 |  |
| BURP21 | Forward | CTACCAAGCCTTCTTCAACTCTTC | 24 | 59.31 | 45.83 | 242 |
|  | Reverse | AAAATGGTGTAGTTCTGCAAACG | 23 | 58.39 | 39.13 |  |
| BURP22 | Forward | GGTTCACCTCAAGGCATAGCA | 21 | 60.34 | 52.38 | 276 |
|  | Reverse | AACAGCATAAGGATAAGGCACAGT | 24 | 60.32 | 41.67 |  |
| BURP23 | Forward | ATGGAGATAAAATGGAAGCTCTTG | 24 | 57.04 | 37.5 | 99 |
|  | Reverse | TCCCAGGCTTGATTCTTAGTGT | 22 | 59.09 | 45.45 |  |
| BURP24 | Forward | GAGCAAACCCATCAAAGGAGA | 21 | 58.2 | 47.62 | 241 |
|  | Reverse | ATGGCAATAGAATACAGCATAAGG | 24 | 56.93 | 37.5 |  |
| BURP25 | Forward | TTTTTACCCCAGAGTATTCTTGCG | 24 | 59.3 | 41.67 | 269 |
|  | Reverse | CGGGTCATTTTTCCACCATTATTA | 24 | 57.54 | 37.5 |  |
| BURP26 | Forward | TTGGCTCAGCTCCAGGTGAAAT | 22 | 62.22 | 50 | 80 |
|  | Reverse | TCAGTTGGCATCATCGGTCC | 20 | 60.11 | 55 |  |
| BURP27 | Forward | TGCTGGGTCCAAAAGTTGTT | 20 | 58.14 | 45 | 139 |
|  | Reverse | ACAGGGAACACTTCCTGGTG | 20 | 59.53 | 55 |  |
| BURP28 | Forward | ATGGGGAGGTTCTATGAAGTGG | 22 | 59.22 | 50 | 118 |
|  | Reverse | TCAACAAAGCGGGATGGTTAT | 21 | 57.92 | 42.86 |  |
| BURP29 | Forward | ATGGGGAGGTTCTATGAAGTGG | 22 | 59.22 | 50 | 118 |
|  | Reverse | TCAACAAAGCGGGATGGTTAT | 21 | 57.92 | 42.86 |  |
| BURP30 | Forward | GGCTCAGGAGATTCGGACAA | 20 | 59.46 | 55 | 240 |
|  | Reverse | AAGTTCAAGAAAAGCAGGATGG | 22 | 57.14 | 40.91 |  |
| BURP31 | Forward | CTCAGGAGATTCGGACAAGGT | 21 | 59.17 | 52.38 | 235 |
|  | Reverse | TTCAAGAAAAGCAGGATGGTTAC | 23 | 57.29 | 39.13 |  |
| BURP32 | Forward | TCCAATGGCGAGGTTCTATG | 20 | 57.38 | 50 | 119 |
|  | Reverse | CAAAAGCAGGATGGTTACGACT | 22 | 58.93 | 45.45 |  |
| BURP33 | Forward | ATGTGGTGGTTCGGACTACTGAG | 23 | 62 | 52.2 | 226 |
|  | Reverse | ACAGCAACCCCTTGATTGATCT | 22 | 58.2 | 45.5 |  |
| BURP34 | Forward | TGCCATTTACGGTTGTCATCA | 21 | 58.22 | 42.86 | 95 |
|  | Reverse | CAAGAGCTTCCATTTTATCTCCAT | 24 | 57.04 | 37.5 |  |
| BURP35 | Forward | TAAAATAGGAGACAACGCAGTGAT | 24 | 58.27 | 37.5 | 220 |
|  | Reverse | AACAGGGACAGTTCCAGGCT | 20 | 61.06 | 55 |  |
| BURP36 | Forward | CTTTTCTGTTTGGTGGAGTGCT | 22 | 59.31 | 45.45 | 236 |
|  | Reverse | CCAGGGTAGAGGTCATGTTGG | 21 | 59.79 | 57.14 |  |
| BURP37 | Forward | CGATGAAGTTCTCAAAGTCAAGCC | 24 | 60.3 | 45.8 | 339 |
|  | Reverse | AACCAGATAAACAAAGACACGCAC | 24 | 58.6 | 41.7 |  |
| BURP38 | Forward | GTCAGGTTCTCAAAGTGAATGGTG | 24 | 60.3 | 45.8 | 72 |
|  | Reverse | GATAAGGAAACAGTAGGGAATGGC | 24 | 60.3 | 45.8 |  |
| BURP39 | Forward | TAAGGACCAATATGTGGTGGATG | 23 | 57.95 | 43.48 | 230 |
|  | Reverse | TGACTTTGAGAATTTCGTAGAGCA | 24 | 58.46 | 37.5 |  |
|  |  |  |  |  |  |  |
|  |  |  |  |  |  |  |
|  |  |  |  |  |  |  |
|  |  |  |  |  |  |  |
